# Disease of the Antrum

**Published:** 1871-12

**Authors:** C. S. Chittenden


### DISEASE OF THE ANTRUM.

BY C. S. CHITTENDEN, D.D.S.

On the 30th of August, 1870, a stout, sturdy Englishman called on me to have the roots of the left superior second bicuspid extracted. The face was most fearfully swollen, the swelling commencing about the orbital edge of the malar bone and extending downward to a point a little below the alæ of the nose, and puffing out in the centre, much as if the half of an egg, cut longitudinally, had been placed under the skin. The surface was very hard and intensely red, the appearance being unlike anything I had ever seen before. I made a good many inquiries, from which I gathered that the swelling first commenced about seven years before, and had given more or less trouble ever since, but had never been as painful or as badly swollen as when he came to me. I also learned that on three or four occasions he had consulted physicians, who had opened the enlargement in the cheek, from which, so far as he knew, there had been no discharge but blood.

The gums were perfectly healthy, there being no inflammation about the roots of the bicuspid even. Suspecting disease of the antrum, I plied him with the usual questions, but failed to elicit anything from him that would lead me to decide positively as to whether the cavity were affected or not, and as I could see no other cause for the trouble, I decided to make an opening into it. For this purpose I extracted the roots, which were removed without difficulty, and then attempted to pass a small drill through the socket of the palatal root, but, as it caused him a good deal of pain,

I desisted for a moment, and then inserted the drill into the socket of the buccal root and gave it two or three turns, when I found it had passed entirely through the bone. I withdrew the drill, expecting to see it followed by a discharge from the swelling; but, as nothing came away, I took up a small probe and passed it through the opening made by the drill, and pressed it up till he asked me to stop, when I found, on measuring, that it had passed up an inch and a half from the edge of the gums. Still there was no discharge. I then took a fine excavator, the shaft of which was bent at an angle of forty-five degrees, and passed that up nearly as far as I had the probe, and rotated it sufficiently to break up any saccular formations within its reach, when fully half a teacupful of very offensive matter was discharged. When all had passed out that would do so, I injected tepid water into the cavity several times till it seemed pretty well cleansed, when I threw in a mixture of iodine and carbolic acid, and placed a tent, saturated in the same mixture, into the opening, and requested him to call the next day.

August 31st.—Patient called, according to appointment. Found the cheek distended nearly as badly as at first. On removing the tent, nearly as much matter was discharged as on the day before.

I filled the cavity with the same mixture, placed another tent in the opening, and requested him to call the next day, which he did. I continued to treat in the same manner for some time, with very little improvement. On probing the cavity carefully, I found that the external wall of the antrum was almost entirely eaten away, so that he could, by sucking, draw the cheek into it, leaving quite a depression on the outside.

On the 8th of September I resolved to try nitric acid, very much diluted. as an injection. Accordingly, I put three or four drops of the acid into a tumbler of water, which diluted it so much that there was only a slight sour taste to it, and injected a syringeful into the cavity every day for a week. From that time he improved rapidly, and in two weeks I discharged him, cured. The opening into the nose was evidently closed, and he positively refused to allow one to be made there. So I could only do the next best thing—form an artificial one into the mouth—which I did.—*Canada Journal of Dental Surgery.*

